# From Context to Care: Rethinking Stigma Detection in Clinical Language Models

**DOI:** 10.2196/82484

**Published:** 2025-10-02

**Authors:** Shefali Haldar, Oliver Bear Don't Walk IV, Sadia Akter

**Affiliations:** 1 Merck Research Laboratories, Merck & Co Inc, 33 Avenue Louis Pasteur, Boston, MA, 02115, United States; 2 Department of Biomedical and Health Informatics, University of Washington, Seattle, WA, United States; 3 Department of Biomedical Sciences, Marshall University, Huntington, WV, United States

**Keywords:** context modeling, health equity, NLP, inclusive language, natural language processing

## Abstract

Natural language processing techniques are useful for identifying stigmatizing language in electronic health records but require careful consideration. This commentary article builds on “*Efficient Detection of Stigmatizing Language in Electronic Health Records via In-Context Learning*” by Chen et al, which highlights the importance of incorporating situational and temporal contexts in annotation and modeling efforts. We emphasize the need for researchers to explicitly articulate their paradigms and positionality, particularly when working with populations disproportionately affected by stigmatizing language. We also explore the differences arising from conflicting preferences across communities about what constitutes destigmatizing language. We discuss participatory and trust-centered approaches for model development to work toward unbiased impact. Such strategies have a crucial role in raising awareness and fostering inclusive health care.

## Introduction

Stigmatizing language in electronic health records contributes to perceptions, influences care decisions, and unintentionally perpetuates socially constructed power dynamics, resulting in bias. There is growing interest in using natural language processing to identify stigma in electronic health records, not only to assess fairness and bias, but also to detect implicit and context-dependent language. Recently, Chen et al [1] demonstrated how large language models can be leveraged to detect stigmatizing language in clinical notes by applying in-context learning, using a custom prompt to guide automated identification of stigma. However, detecting stigma is not only a technical challenge, but also a social and ethical one.

This commentary builds on their work, outlining the key considerations for future research. We emphasize the importance of contextual modeling to identify stigmatizing language that subtly reinforces bias. We also emphasize researcher self-awareness and contextual understanding, especially when working with marginalized communities. We discuss the differences in language across clinicians, patients, caregivers, and communities and its impact on model fairness. Finally, we advocate for participatory, equity-centered approaches that foster trust and accountability in the development of bias detection tools.

## Considering the Context and Mode of Implicit Stigmatizing Language

Annotating a training/benchmarking corpus for stigmatizing language can help to identify subtle and contextually dependent instances (eg, homonyms). However, a focus on explicit mentions may miss some forms of implicit stigmatizing language. For example, “Patient discussed using tobacco in cultural ceremonies and has been advised on the dangers of smoking to her health” may not be flagged as stigmatizing with a bias toward explicit mentions. However, one could argue that this sentence stigmatizes both the patient’s beliefs and a cultural practice by labeling this tobacco use as potentially unhealthy.

The authors acknowledge this limitation and reveal an opportunity for future work to explore implicit or implied stigma, an inherently difficult task that responds to 3 central features of stigmatizing language: subtle, implicit, and highly contextual.

Addressing these features requires a broader contextual window around a given sentence and consideration of situational, relational, and temporal factors. The authors used a 15-word symmetric window on either side of the input sentence, suggesting this can be expanded with improved modeling approaches and additional resources.

However, understanding subtle instances of stigmatizing language may require context that lies beyond the clinical note. These include multimodal elements such as tone, body language, identities, and relationship dynamics between clinician and patient. Capturing this richer context may require incorporating audio, video, or self-reported data and a reflexive understanding of how power relationships shape clinical interactions. These added data sources require ethical considerations beyond current protected health information guardrails; for example, can one objectively identify tone and body language? How and when should information be extracted from video and audio if it was not deemed clinically relevant by a clinician? How can patients request to edit their video or audio data?

## Acknowledging the Positionality of Researchers

The ontological and epistemological leanings of the research team can influence assumptions about stigmatizing language in clinical notes. Research can be strengthened and the audiences better informed if research teams outline their guiding paradigms and assumptions. Additionally, research into stigmatizing language could benefit from positionality statements where factors that influence decision-making are acknowledged, better enabling the identification of limitations and future directions.

For example, was the research process informed by views from patients from specific populations, cultural backgrounds, socioeconomic status, or groups more likely to experience stigmatizing language? Similar to outlining methodological assumptions and approaches, describing what positions influenced the research process can improve its rigor, transparency, and application.

## Accounting for Patient Perspectives and Conflicting Values

Chen et al discuss the importance of transparent communication about the model’s purpose and recognize the risk of enforcing stereotypes of historically marginalized groups. Strategies such as decision-support alerts and prompted artificial intelligence scribing [2] aim to encourage reflection about this risk among clinical teams.

However, we must account for the patients and caregivers affected by the model’s outputs and resulting care decisions. Although the authors acknowledge variations in how stigmatizing language materializes in different types of clinical documentation and health contexts, it is equally important to recognize tensions between what clinicians, patients, caregivers, and communities deem an appropriate health care interaction. What one group interprets as destigmatizing language may be perceived differently by another group. Within a group, individuals can have conflicting preferences for what language best reflects their shared identity.

Plurality—the concept that there is no “correct” form of destigmatized language—requires us to consider alternative and potentially contradictory values when refining the sensitivity of models [3,4]. In recognizing the limitations of a binary stigmatizing versus destigmatizing framework, Chen et al point us to avenues for future work: (1) elicit these contradictory values and explore how model parameters should be tailored to account for and address each community’s unique challenges and (2) examine ethical ways to engage communities in this work while minimizing privacy risks.

## Building Trust and Equity

Given nuanced views of what is considered destigmatized language, researchers must leverage strategies to build trust and center marginalized communities as they develop and implement models. For example, recent human-computer interaction work has outlined concrete steps to address racial gaps in participatory methods, providing guidance for health equity–focused artificial intelligence design, implementation, and decision-making [5]. Theoretical and practical frameworks can provide an equity lens for technology research and implementation [6,7].

Applying this knowledge can help avoid historical patterns of research exploitation while ensuring the model’s purpose, outputs, and implementation are driven by and aligned with the values of the communities they are intended to benefit.

## Conclusion

Standalone technologies cannot completely eliminate stigma and bias in health care interactions. However, with evolving social norms and forward cultural progress, these technologies offer critical ways to minimize harmful impact and address root causes of health inequity. Importantly, Chen et al affirm that the intention of bias-detection models is not to surveil or punish the use of stigmatizing language; instead, the goal is to increase bias awareness in the daily practice of delivering and receiving care. With greater awareness, we can remove barriers to health equity and achieve improved health outcomes.
